# Characterization of Catecholaldehyde Adducts with Carnosine and l-Cysteine Reveals Their Potential as Biomarkers of Catecholaminergic Stress

**DOI:** 10.1021/acs.chemrestox.1c00153

**Published:** 2021-09-10

**Authors:** Rachel A. Crawford, Ettore Gilardoni, T. Blake Monroe, Luca Regazzoni, Ethan J. Anderson, Jonathan A. Doorn

**Affiliations:** † Department of Pharmaceutical Sciences & Experimental Therapeutics, College of Pharmacy, University of Iowa, 180 South Grand Avenue, Iowa City, Iowa 52242, United States; ‡ Department of Pharmaceutical Sciences, University of Milan, Via L. Mangiagalli 25, Milan 20133, Italy; § Fraternal Order of Eagles Diabetes Research Center, University of Iowa, Iowa City, Iowa 52242, United States

## Abstract

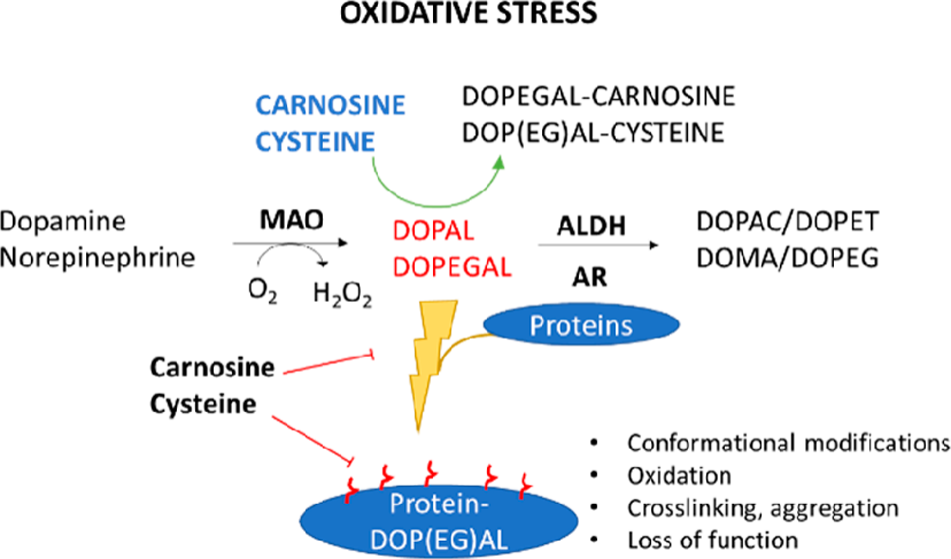

Monoamine oxidase (MAO) catalyzes the oxidative deamination of dopamine and norepinephrine to produce 3,4-dihydroxyphenylacetaldehyde (DOPAL) and 3,4-dihydroxyphenylglycolaldehyde (DOPEGAL), respectively. Both of these aldehydes are potently cytotoxic and have been implicated in pathogenesis of neurodegenerative and cardiometabolic disorders. Previous work has demonstrated that both the catechol and aldehyde moieties of DOPAL are reactive and cytotoxic via their propensity to cause macromolecular cross-linking. With certain amines, DOPAL likely reacts via a Schiff base before oxidative activation of the catechol and rearrangement to a stable indole product. Our current work expands on this reactivity and includes the less-studied DOPEGAL. Although we confirmed that antioxidants mediated DOPAL’s reactivity with carnosine and *N*-acetyl-l-lysine, antioxidants had no effect on reactivity with l-cysteine. Therefore, we propose a non-oxidative mechanism where, following Schiff base formation, the thiol of l-cysteine reacts to form a thiazolidine. Similarly, we demonstrate that DOPEGAL forms a putative thiazolidine conjugate with l-cysteine. We identified and characterized both l-cysteine conjugates via HPLC-MS and additionally identified a DOPEGAL adduct with carnosine, which is likely an Amadori product. Furthermore, we were able to demonstrate that these conjugates are produced in biological systems via MAO after treatment of the cell lysate with norepinephrine or dopamine along with the corresponding nucleophiles (i.e., l-cysteine and carnosine). As it has been established that metabolic and oxidative stress leads to increased MAO activity and accumulation of DOPAL and DOPEGAL, it is conceivable that conjugation of these aldehydes to carnosine or l-cysteine is a newly identified detoxification pathway. Furthermore, the ability to characterize these adducts via analytical techniques reveals their potential for use as biomarkers of dopamine or norepinephrine metabolic disruption.

## Introduction

Monoamine oxidase (MAO)-catalyzed deamination of norepinephrine (NE) and dopamine (DA) yields 3,4-dihydroxyphenylglycolaldehyde (DOPEGAL) and 3,4-dihydroxyphenylacetaldehyde (DOPAL), respectively, as well as H_2_O_2_.^1^ Under physiological conditions, these catecholaldehyde metabolites are detoxified primarily by aldehyde dehydrogenase (ALDH) to their corresponding carboxylic acid or by aldose reductase (AR) to their corresponding alcohol.^2^ Under conditions of oxidative stress, a multilevel dysregulation of catecholamine metabolism occurs, resulting in an accumulation of the aldehyde intermediates.^1^ Aberrant production of these aldehydes, which are highly reactive and cytotoxic, has been implicated in disease etiopathology.^1,3^ This is often referred to as the “catecholaldehyde hypothesis”.^1^ DOPAL has been studied for its role in the pathogenesis of Parkinson’s disease: DOPAL can covalently modify proteins,^35,4,5^ generate reactive oxygen species and radicals,^6^ and promote oligomerization of α-synuclein, a hallmark of Parkinson’s.^7,8^ Importantly, both DOPAL^9^ and DOPEGAL^10^ have recently been implicated in Alzheimer’s disease pathogenesis due to their potent activation of asparagine endopeptidase, the enzyme involved in amyloid precursor protein and Tau accumulation. DOPEGAL has also been postulated to be involved in the etiology of cardiovascular diseases,^11^ as our group has observed formation of this catecholaldehyde in mitochondrial preparations of human heart, where it has been linked to disruption of oxidative phosphorylation in diabetes patients.^12,13^ Though a direct pathogenic link between DOPEGAL and cardiovascular diseases remains to be established, both clinical and experimental studies have identified MAO as playing a pathological role in cardiac injury from ischemia, diabetes, and hypertension.^14−17^

**Figure 1:**
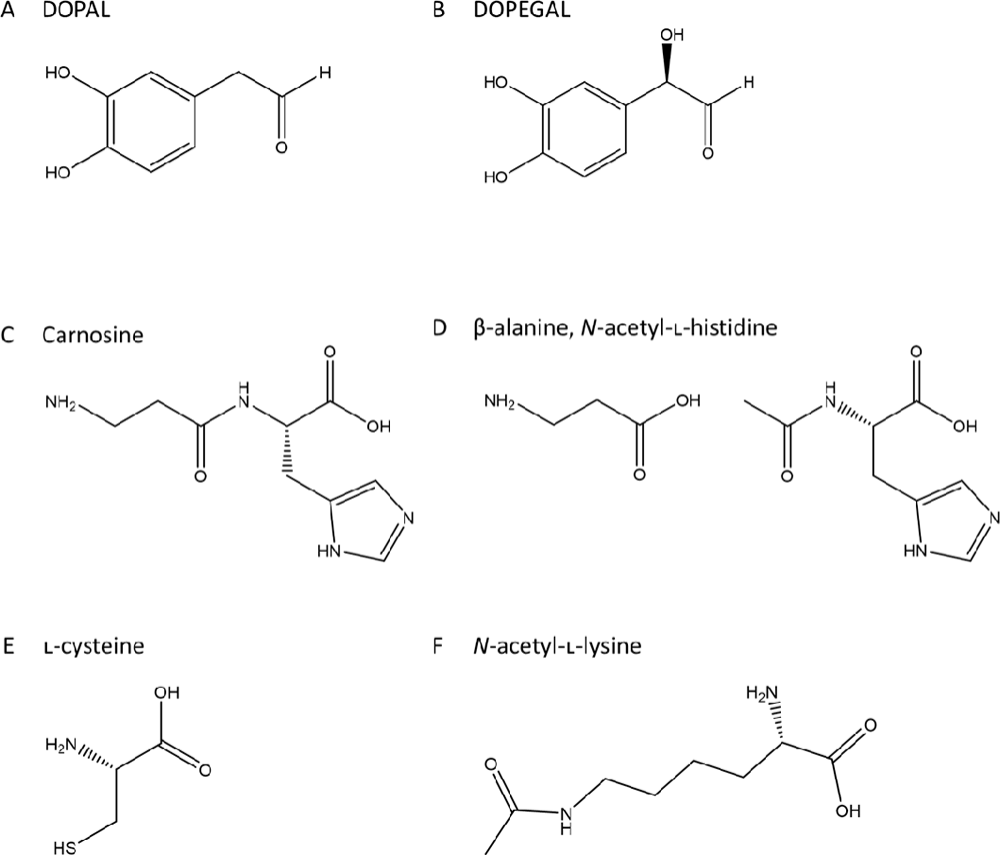
Summary of the compounds investigated in this report. The two biogenic aldehydes, DOPAL (A) and DOPEGAL (B), are unique in their possession of both an aldehyde and a catechol moiety. Carnosine (C) is a dipeptide composed of the amino acids β-alanine (D) and l-histidine, though the acetylated version of l-histidine is used in this report to simulate a peptide bond blocking the amino group. Other scavengers investigated include l-cysteine (E) and *N*-acetyl-l-lysine (F).

The high cytotoxicity of DOPEGAL and DOPAL is caused by the reactivity of both the catechol and aldehyde constituents.^18^ These unique structures, along with a summary of the compounds investigated in this report, can be viewed in Figure 1. As previously reported, catecholaldehydes form covalent, stable adducts with protein amines such as lysine and other nucleophilic molecules.^6,19^ This causes permanent modification of protein structure and function.^4,20^ Our group has previously investigated the ability of known nucleophiles, such as the dipeptide carnosine and amino acid l-cysteine, to scavenge DOPAL and DOPEGAL and hence protect cellular proteins from modification.^13^ Our previous work showed carnosine and l-cysteine were able to sequester DOPAL in vitro, but information on reactivity of DOPEGAL is very limited^21^ due to its commercial unavailability and difficult synthesis.^13,19^ In this report, we further characterize the reactivity of carnosine and l-cysteine with both DOPAL and DOPEGAL biochemically and in a cellular matrix. Adducts of carnosine with DOPEGAL and l-cysteine with DOPEGAL and DOPAL were identified and characterized via mass spectrometry. We were also able to confirm that this adduct formation still occurs in a cellular matrix, where other nucleophiles or concomitant reactions can compete. Most importantly, we demonstrate that MAO activity is necessary and sufficient for conjugate formation in a cell lysate, suggesting that the formation of these adducts may be novel detoxification pathways for catecholaldehydes and that the resultant conjugates could be biomarkers for neuro or cardiac injury.

## Experimental Procedures

### Chemicals

All chemicals were purchased from Sigma-Aldrich (St. Louis, MO, U.S.A.) unless otherwise noted. DOPAL was purchased from Cayman Chemical (Ann Arbor, MI, U.S.A.). Recombinant MAO-A was purchased from Corning (Glendale, AZ, U.S.A.). Trypsin 0.25% solution, DMEM, F12, 1× PBS, Opti-MEM, 100 mM sodium pyruvate, MEM nonessential amino acids, fetal bovine serum, and penicillin/streptomycin were purchased from Gibco (Grand Island, NY, U.S.A.).

### DOPEGAL Synthesis

DOPEGAL was synthesized as reported by Nilsson et al.^22^ Briefly, NE at a final concentration of 2 mM was incubated with 2.25 μg/mL of recombinant MAO-A in 10 mM potassium phosphate buffer (pH 7.4) and 5 mM sodium bisulfite for 10 h at 30 °C in the presence of oxygen. The incubation was stopped by centrifugation at 100,000*g* for 30 min and the supernatant stored at −80 °C prior to use. The sodium bisulfite addition stabilizes DOPEGAL by forming a hemithioacetal; this stabilization creates easier handling but is reversible and therefore suitable for studying DOPEGAL reactivity.

### Cell Lysate Preparation

All experiments using rodent models were conducted with approval from the Institutional Animal Care and Use Committee at the University of Iowa. C57/Bl6J mice were used for these experiments (Jackson Laboratories). Animals were housed in temperature- and light-controlled conditions with free access to food and water. Neonatal hearts were harvested from D1–D3 pups, and primary cardiac fibroblasts were prepared via enzymatic digestion using a proprietary enzyme mix (Pierce Primary Cardiomyocyte Isolation Kit, Thermo Fisher, Waltham, MA, U.S.A.). Following digestion, a sequential plating method was used to select for fibroblasts. Fibroblasts were cultured as a monolayer in DMEM/F12 (1/1, v/v) containing 10% fetal bovine serum at 37 °C in a humidified atmosphere of 5% CO_2_.

SH-SY5Y human neuroblastoma cells were obtained from American Type Culture Collection (Manassas, VA, U.S.A.). These cells were grown in Opti-MEM supplemented with 10% fetal bovine serum, 1% MEM-nonessential amino acids, 1% penicillin/streptomycin, and 1 mM sodium pyruvate at 37 °C in a humidified atmosphere of 5% CO_2_. All experiments on these cells were performed prior to passage 30.

At confluence, cells were harvested with trypsin solution, transferred to a tube, and centrifuged at 300*g* for 5 min. The pellet obtained was washed twice with PBS to minimize residual trypsin. Cell pellets were stored at −80 °C (inducing enzyme deactivation) prior to analysis or freshly used (maintaining enzyme activity).

Cell pellets were resuspended in 300 μL of 1 mM PBS and lysed by sonication with a Sonic Dismembrator (Fisher Scientific, Pittsburgh, PA, U.S.A.). Cell lysate was centrifuged at 10,000*g* for 10 min at 4 °C, supernatant was collected, and the protein content (mg/mL) was measured via BCA assay.

### Analysis of Carnosine and Cysteine Sequestering Activity

#### Biochemical Analysis:

Carnosine or l-cysteine was incubated with DOPEGAL or DOPAL at a final concentration of 1 mM and 100 μM, respectively, in 10 mM PBS (pH 7.4) at 37 °C, which represents a 10:1 ratio for nucleophile/electrophile. For HPLC-MS analysis, reactions were quenched at 4 h by diluting 1:5 with 0.1% FA and directly analyzed via HPLC-MS or stored at −20 °C. For HPLC-PDA analysis, DOPEGAL or DOPAL were incubated with a variety of nucleophiles (carnosine, l-cysteine, *N*-acetyl-l-lysine, *N*-acetyl-l-histidine, and β-alanine) at a final concentration of 100 μM and 1 mM, respectively. Experiments involving carnosine or *N*-acetyl-l-lysine (NAL) were repeated in the presence of 1 mM NaCNBH_3_ or *N*-acetyl-l-cysteine (NAC), which represents a concentration of 1:1 nucleophile/NaCNBH_3_/NAC and 1:10 DOPAL/DOPEGAL/NaCNBH_3_/NAC. Aliquots were sampled at the desired time points and diluted 1:1 with an aqueous solution containing 1% acetonitrile (ACN) and 1% trifluoroacetic acid (TFA) to stop the reaction. Samples were directly analyzed with the HPLC-PDA system or stored at −20 °C.

#### Inactive Cell Lysate:

Carnosine or l-cysteine was incubated with DOPEGAL or DOPAL at a final concentration of 1 mM and 100 μM, respectively, in 100 μg/mL cell lysate (diluted in 1 mM PBS, pH 7.4) at 37 °C, which represents at 10:1 ratio of reactants. Aliquots were sampled at the desired time points and deproteinized by adding TCA at a final concentration of 5% v/v. Deproteinized samples were centrifuged at 10,000*g* for 10 min at 4 °C. The supernatant was stored at −20 °C or diluted 1:5 with 0.1% FA solution prior to analysis with the HPLC-MS system.

#### Recombinant MAO-A Solution:

Carnosine was incubated with NE at a final concentration of 770 μM and 7.70 mM in 10 mM phosphate buffer with 2.25 μg/mL of recombinant MAO-A at 30 °C in the presence of oxygen. The incubation was stopped by centrifugation at 100,000*g* for 30 min. Supernatant was diluted 1:8 with an aqueous solution containing 1% ACN and 1% TFA and directly analyzed with the HPLC-PDA system or stored at −20 °C. This reaction was repeated in the presence of 1 μM clorgyline (i.e., an MAO-A inhibitor).

#### Active Cell Lysate:

Carnosine or l-cysteine was incubated with NE or DA at the final concentration of 1 mM and 50 μM, respectively (20:1 ratio), in 100 μg/mL active cell lysate (diluted in 1 mM PBS, pH 7.4) at 37 °C. Aliquots were sampled at 0 and 24 h and deproteinized by adding TCA at a final concentration of 5% v/v. Deproteinized samples were centrifuged at 10,000*g* for 10 min at 4 °C. The supernatant was diluted 1:5 with 0.1% FA solution prior to analysis with the HPLC-MS system. The reactions were repeated in the presence of 1 μM clorgyline and 1 μM selegiline (i.e., MAO-A and MAO-B inhibitors). To control for the possibility of selegiline or clorgyline reacting with the aldehydes, a reaction of 50 μM DOPEGAL or DOPAL and 1 mM carnosine or l-cysteine was incubated in 10 mM PBS (pH 7.4) in the absence or presence of 1 μM selegiline or clorgyline and evaluated via HPLC-PDA.

### HPLC-PDA Analysis

DOPAL concentration was measured via a 1200 series Agilent HPLC Capillary HPLC system with a photodiode array detector (PDA) at 280 nm (Agilent Technologies, Santa Clara, CA, U.S.A.). Chromatographic separation was carried out with a Phenomenex C18 Luna column (150 × 1 mm, particle size 5 μM) using an isocratic method with a mobile phase of 97% HPLC-grade water, 3% HPLC-grade ACN, and 0.1% TFA with a flow rate of 50 μL/min. Acquisition and analysis were performed using ChemStation V.0.0.1.52 (Agilent Technologies, Santa Clara, CA, U.S.A.). Data were visualized using GraphPad Prism (GraphPad Software, San Diego, CA, U.S.A.). Chromatograms presented are representative of at least three trials.

### HPLC-ESI-QTOF

HPLC-MS analysis was performed on an Agilent 6530 quadrupole-time-of-flight mass spectrometer interfaced with a 1260 Series Agilent capillary HPLC system (Agilent Technologies, Santa Clara, CA, U.S.A.). Samples were injected at a volume of 5 μL. Chromatographic separation was carried out at a flow rate of 15 μL/min with a Zorbax column (150 × 0.5 mm; particle size 5 μm) with mobile phase A (UHPLC grade H_2_O, 0.1% FA) and B (UHPLC grade ACN, 0.1% FA) in the following gradient (A/B): 0 min (97:3) → 1 min (97:3) →10 min (40:60) → 13 min (5:95) → 17 min (5:95) → 17.01 min (97:3) → 30 min (97:3).

Analytes were eluted into a dual ESI jet stream source. Ionization was carried out in positive ion mode with the following source parameters: capillary voltage 3500 V; nebulizer gas 35 psig; sheath gas 10 L/min; sheath temperature 320 °C; drying gas 5 L/min; gas temperature 300 °C; fragmentor 175 V. The scan range was 200–700 *m*/*z* with a 1 spectra/s scan time. MS/MS fragmentation analysis was carried out using a targeted method based on the full spectrum scan (i.e., with desired precursor ion and retention time window) with 100 ms as the scan time and 20 V as the collision energy. Data were acquired using MassHunter LC/MS Data Acquisition v.B.05.01, and data analysis was performed with MassHunter Qualitative Analysis v. B.06.00 (Agilent, U.S.A.) and GraphPad Prism (GraphPad Software, San Diego, CA, U.S.A.). Chromatograms and spectra presented are representative of at least three trials.

## Results

### DOPAL Reactivity with Amines Decreases in the Presence of Antioxidants

**Figure 2:**
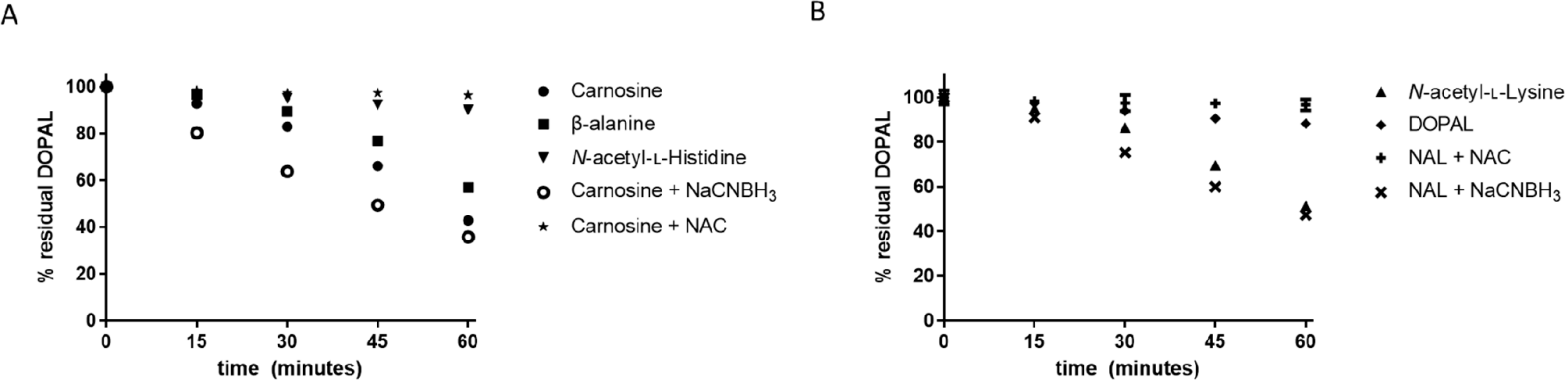
HPLC-PDA analysis of the reaction of DOPAL with carnosine or *N*-acetyl-l-lysine (NAL) (*n* = 3). Reactivity with NAL, carnosine, and β-alanine (A) suggests that DOPAL reacts with the amine constituent. These data also suggest that the mechanism of reactivity of DOPAL with carnosine and NAL requires oxidative activation of the catechol ring. (A) DOPAL was reacted with the dipeptide carnosine or its amino acid constituents, *N*-acetyl-l-histidine and β-alanine. The decrease in % residual DOPAL corresponds to the reactive consumption of DOPAL in the varying conditions applied. DOPAL appears to react with carnosine and with β-alanine but has little reactivity with *N*-acetyl-l-histidine, suggesting that β-alanine is the amino acid primarily responsible for the reactivity of carnosine with DOPAL. DOPAL’s % reaction with carnosine was increased with the addition of the reducing agent NaCNBH_3_ but decreased in the presence of antioxidant NAC. (B) DOPAL was reacted with NAL. Reactivity with NAL increases with the addition of a reducing agent but decreases in the presence of an antioxidant.

DOPAL reactivity with NAL was assessed by measuring the decrease in DOPAL concentration over time via HPLC-PDA (*n* = 3). Figure 2B demonstrates the changes in DOPAL concentration with and without NAL, and in the presence of an antioxidant (NAC) or reducing agent (NaCNBH_3_). The initial ratio of NAL/DOPAL was 10:1. After 60 min, DOPAL dropped to 88.2 ± 1.31% of the initial concentration in the control mixture (no reactant). When incubated with NAL, DOPAL concentration dropped to 51.4 ± 1.12%. Interestingly, this concentration dropped even further, down to 47.4 ± 1.22%, when DOPAL was incubated with NAL in the presence of NaCNBH_3_. In contrast, reactivity of DOPAL and NAL decreased in the presence of NAC: 96.7 ± 2.48% of initial DOPAL concentration remained after 60 min. This reaction also demonstrates that DOPAL is stabilized in the presence of NAC + NAL compared to the control.

Similarly, DOPAL reactivity with carnosine was investigated, as shown in Figure 2A. After 60 min, DOPAL dropped to 42.8 ± 1.13% of its initial concentration when incubated with carnosine (*n* = 3). The presence of an antioxidant or reducing agent demonstrated reactivity changes similar to those with NAL. Reactivity was increased in the presence of NaCNBH_3_, with only 35.8 ± 0.80% DOPAL remaining after 60 min. In contrast, reactivity decreased in the presence of NAC, with 96.2 ± 0.68% of DOPAL remaining. To further investigate the reactivity of DOPAL with carnosine, DOPAL was incubated with the two amino acid constituents of carnosine, β-alanine and *N*-acetyl-l-histidine, individually. After 60 min with β-alanine, which contains the amine group of carnosine, DOPAL concentrations dropped to 56.9 ± 0.35% of the original. With *N*-acetyl-l-histidine, DOPAL only decreased to 90.2 ± 1.24% of initial (note that the control DOPAL dropped to 88.2 ± 1.31% in Figure 2B).

### DOPAL Reactivity with l-Cysteine Does Not Decrease in the Presence of Antioxidants

**Figure 3:**
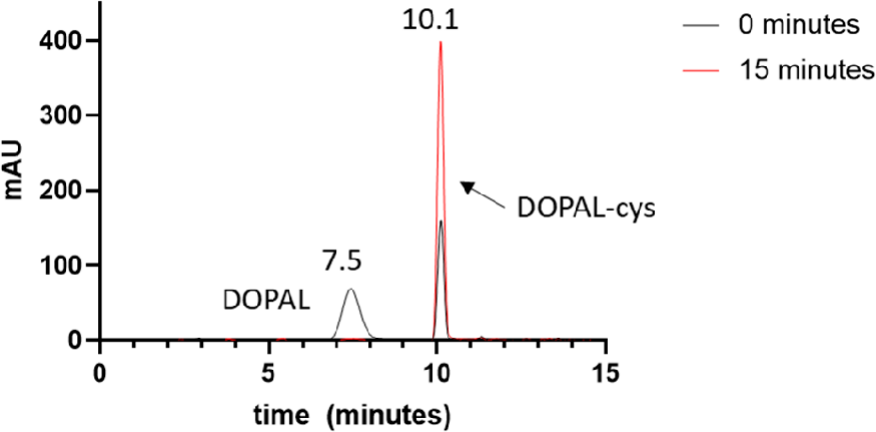
HPLC-PDA analysis of the reaction of DOPAL with l-cysteine. At time ∼0, the conjugate peak is already observed due to the quick reaction of the two compounds. After 15 min, the DOPAL peak disappears and the intensity of the presumed conjugate peak increases, implying completion of reaction and formation of DOPAL–cys.

DOPAL reactivity with l-cysteine was investigated via HPLC-PDA. Control DOPAL had a retention time (*t*_R_) of ∼7.5 min (data not shown). This peak can be observed at time 0 in our reaction sample; this peak flattened after 15 min of incubation with l-cysteine (Figure 3). In contrast, a peak unique to the reaction sample (*t*_R_ ∼ 10.1 min) increased after 15 min, implying complete consumption of DOPAL by l-cysteine and formation of a new product. This peak is already present at reaction time 0, indicating near instantaneous reaction of DOPAL and l-cysteine. Furthermore, the peak at *t*_R_ 10.1 min does not decrease when DOPAL and l-cysteine are reacted in the presence of NAC (data not shown), implying a different mechanism of reactivity for l-cysteine than the other tested nucleophiles.

### DOPEGAL Reactivity with Carnosine and l-Cysteine

**Figure 4:**
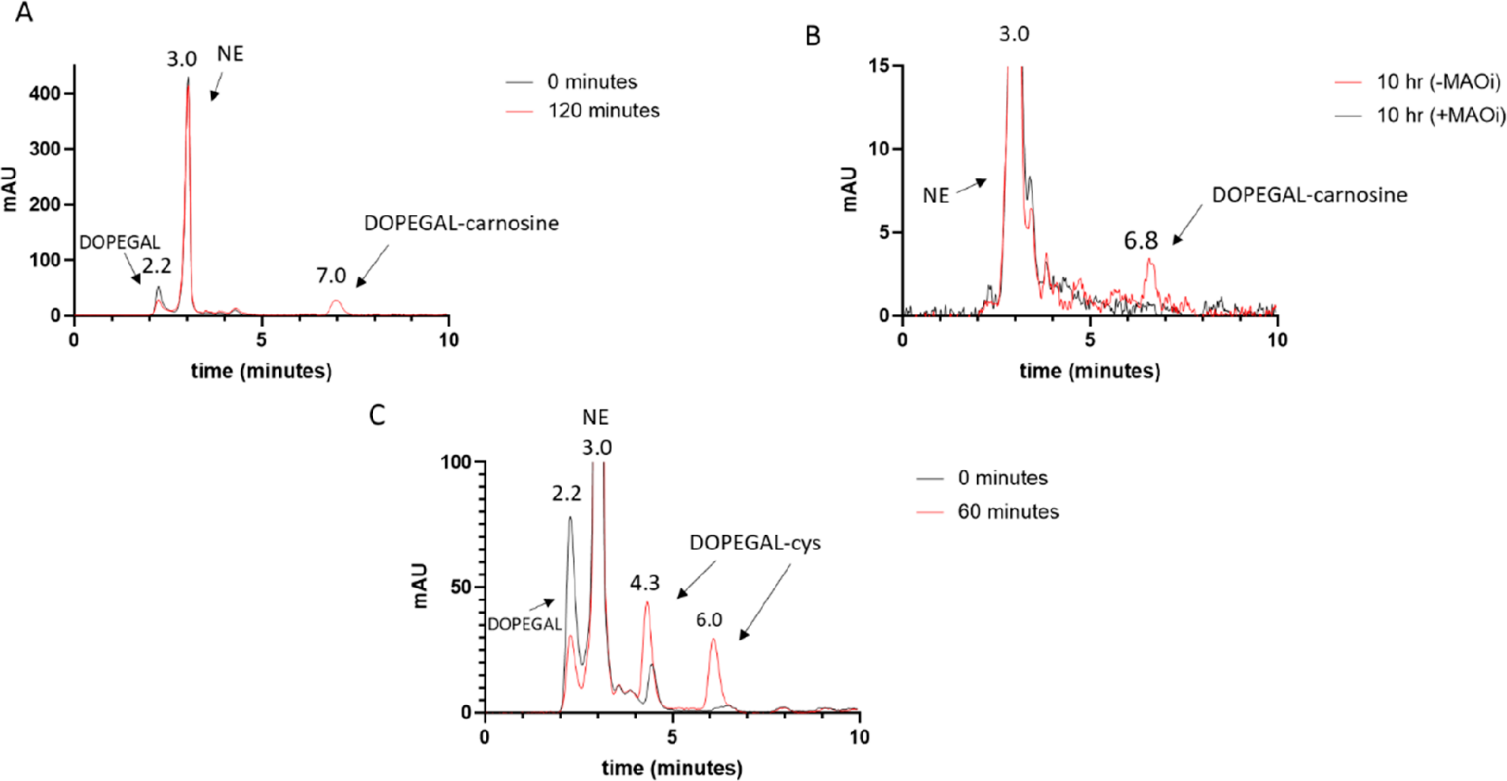
HPLC-PDA (280 nm) analysis of the reaction of DOPEGAL with carnosine or l-cysteine. DOPEGAL readily reacts with both scavengers. (A) Carnosine was reacted with DOPEGAL. After 2 h of incubation, a new peak is formed at a retention time of ∼7.0 min, presumably the conjugate of carnosine and DOPEGAL. This peak is not observed at time 0. (B) Carnosine was incubated with NE and recombinant MAO-A. After 10 h, a peak is observed corresponding with the retention time of carnosine-DOPEGAL in A. This peak does not appear when clorgyline, an MAO-A inhibitor, is present. (C) DOPEGAL was reacted with l-cysteine. At time 0, a DOPEGAL, NE, and two potential conjugate peaks are visible. After 60 min, the two unassigned peaks increase in intensity and the DOPEGAL peak decreases, implying the formation of multiple DOPEGAL–cys products.

The reactivity of DOPEGAL with carnosine and l-cysteine was investigated via HPLC-PDA. While we have published previous work demonstrating the reactivity of DOPAL with a variety of nucleophiles,^6,13,19^ these data did not include the reactivity of DOPEGAL. DOPEGAL was synthesized via Nilsson’s method using NE;^22^ consequently, excess NE is observed in these reactions (Figure 4, *t*_R_ ∼ 3.0). Note that NE does not appear to be consumed over time (Figure 4).

After 2 h, the reaction of carnosine and DOPEGAL formed a new peak that can be observed at *t*_R_ ∼ 7 (Figure 4A), which was not observed when carnosine was incubated with NE (data not shown). A decrease in the DOPEGAL peak (*t*_R_ ∼ 2.2) can be observed over time. To further investigate the reactivity of carnosine with DOPEGAL, we also incubated carnosine with NE and MAO-A in the absence or presence of clorgyline, an MAO-A inhibitor (MAOi). In the absence of clorgyline, a new peak was formed at *t*_R_ ∼ 6.8 (Figure 4B). DOPEGAL was also reacted with l-cysteine. This reaction yielded at least two new peaks (Figure 4C), at *t*_R_ ∼ 4.3 and ∼6.0 min, which were not present in a reaction of l-cysteine and NE (data not shown). Furthermore, a decrease in the DOPEGAL peak (*t*_R_ ∼ 2.2) can be observed over time.

### Characterization of DOPEGAL and DOPAL Conjugates

**Figure 5:**
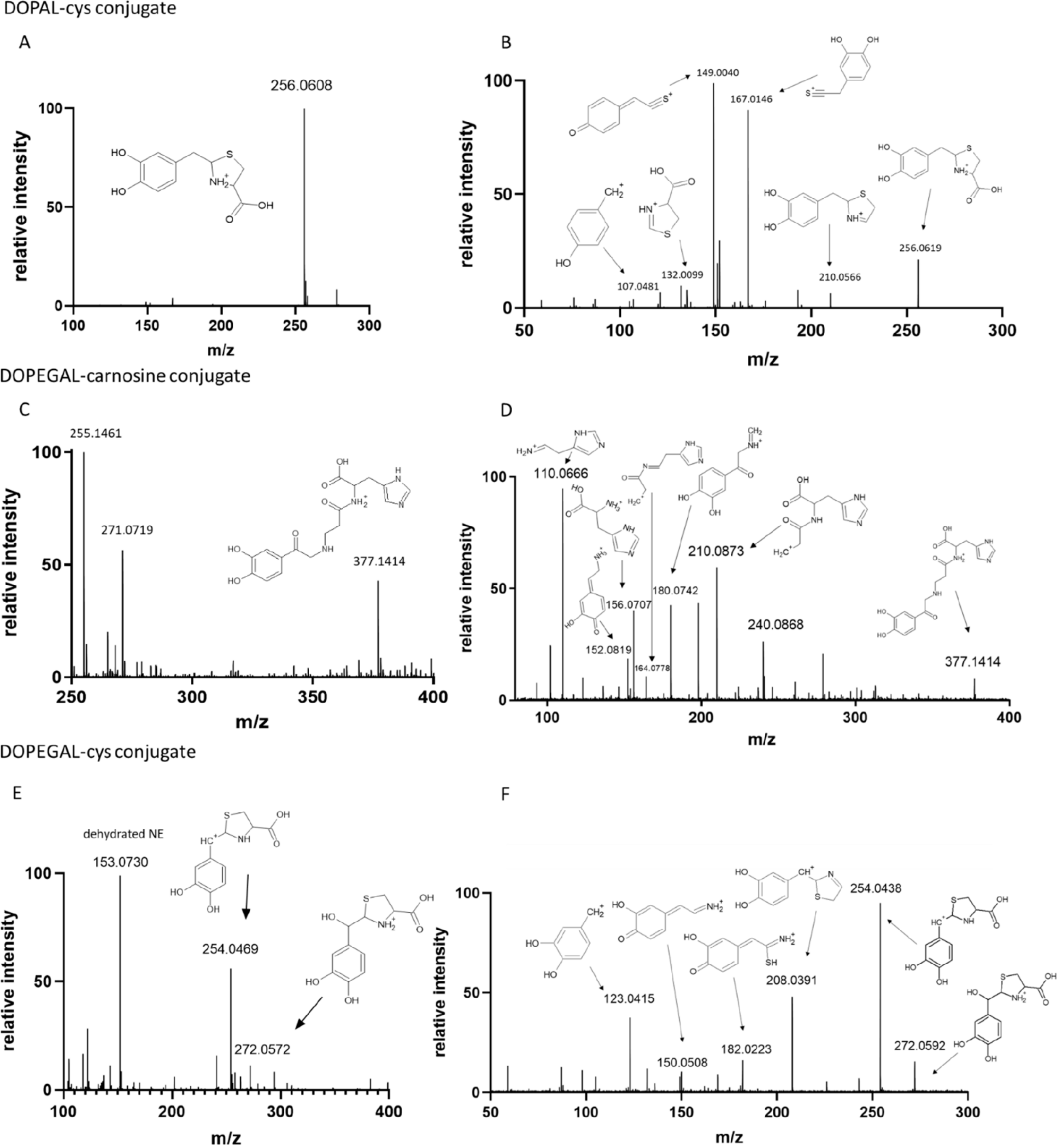
HPLC-ESI-MS analysis of the biochemical reaction of DOPAL with l-cysteine and DOPEGAL with carnosine or l-cysteine. The DOPAL–carnosine conjugate was not observable. (A,B) HPLC-MS analysis of a biochemical reaction of DOPAL and l-cysteine. The full scan spectrum of DOPAL–cys (A) can be observed with an experimental *m*/*z* at 256.0608 (theoretical *m*/*z* = 256.0638). The MS/MS spectrum (B) shows the fragmentation pattern with putative structure assignments, supporting our proposed conjugate structure. (C,D) HPLC-MS analysis of a biochemical reaction of DOPEGAL and carnosine. The full spectrum of DOPEGAL–carnosine (C) can be observed with an experimental *m*/*z* at 377.1469 (theoretical *m*/*z* = 377.1445). The MS/MS spectrum (D) shows the fragmentation pattern with putative structure assignments, supporting our proposed conjugate structure. (E,F) HPLC-MS analysis of a biochemical reaction of DOPEGAL and l-cysteine. The full spectrum of DOPEGAL–cys (E) can be observed with an experimental *m*/*z* at 272.0572 and *m*/*z* 254.0469 (theoretical *m*/*z* = 272.0587, 254.0482). Two major peaks result because the DOPEGAL–cys conjugate easily dehydrates, resulting in a decrease in mass of ∼18 amu. The MS/MS spectrum (F) shows the fragmentation pattern with putative structure assignments, supporting our proposed conjugate structure. Though multiple DOPEGAL–cys conjugates are conceivable (see Figure 3C), the 254.04 *m*/*z* was a base peak in the BPC; other DOPEGAL–cys conjugates were are not easily identifiable or not in great abundance.

After observing new compound formation via HPLC-PDA, we then characterized these compounds via HPLC-MS. We did not identify a DOPAL adduct with either NAL or carnosine, though the HPLC-PDA data support reactivity. We did identify a DOPAL adduct with l-cysteine (DOPAL–cys; Figure 5A) with an [M + H]^+^ peak at 256.0608 *m*/*z* (theoretical [M + H]^+^ = 256.0638 *m*/*z*, accuracy = −11.71 ppm). A fragmentation experiment was conducted to characterize this adduct. We suggest a thiazolidine conjugate and the putative fragments are reported in Figure 5B.

We were able to identify DOPEGAL adducts with both carnosine (DOPEGAL–carnosine; Figure 5C) and l-cysteine (DOPEGAL–cys; Figure 5E). Our findings align with prior reports of an Amadori product for DOPEGAL and carnosine,^21^ with an [M + H]^+^ at 377.1469 *m*/*z* (theoretical [M + H]^+^ = 377.1445 *m*/*z*, accuracy = 6.36 ppm). The structure was further characterized via MS/MS fragmentation analysis, and the putative fragment structures are reported in Figure 5D. Like with DOPAL–cys, we also suggest a thiazolidine conjugate for DOPEGAL–cys, which appears at an [M + H]^+^ peak of 272.0572 *m*/*z* (theoretical [M + H]^+^ = 272.0587 *m*/*z*, accuracy = −5.51 ppm). Our fragmentation data support a thiazolidine structure (Figure 5F), as was previously reported.^21^ Similarly, we observed dehydration of the DOPEGAL–cys adduct (254.0469 *m*/*z*). The dehydrated molecular ion [M – OH]^+^ is more abundant than the intact molecular ion. The structure was further characterized via MS/MS analysis, and the putative fragment structures are reported in Figure 5F.

### Identification of Conjugates in a Biological System

Next, we examined if the reactivity of carnosine with DOPEGAL and l-cysteine with DOPEGAL or DOPAL is maintained in a biological environment where numerous other nucleophiles may compete. Inactive cell lysate (lysate that had been frozen with reduced enzymatic activity) was used to evaluate reactivity of DOPAL and DOPEGAL in an environment with concurrent nucleophilic reactions but with little to no metabolic transformation as a variable. For DOPAL reactivity, SH-SY5Y neurons were used. SH-SY5Y cells are commonly used to explore Parkinson’s pathology.^23^ For DOPEGAL reactivity, primary cardiac fibroblasts were used, as DOPEGAL has been suggested to play a role in pathogenesis of cardiac diseases^11^ and because our concurrent study has shown that carnosine mitigated the profibrotic effects of DOPEGAL in these cells.

**Figure 6:**
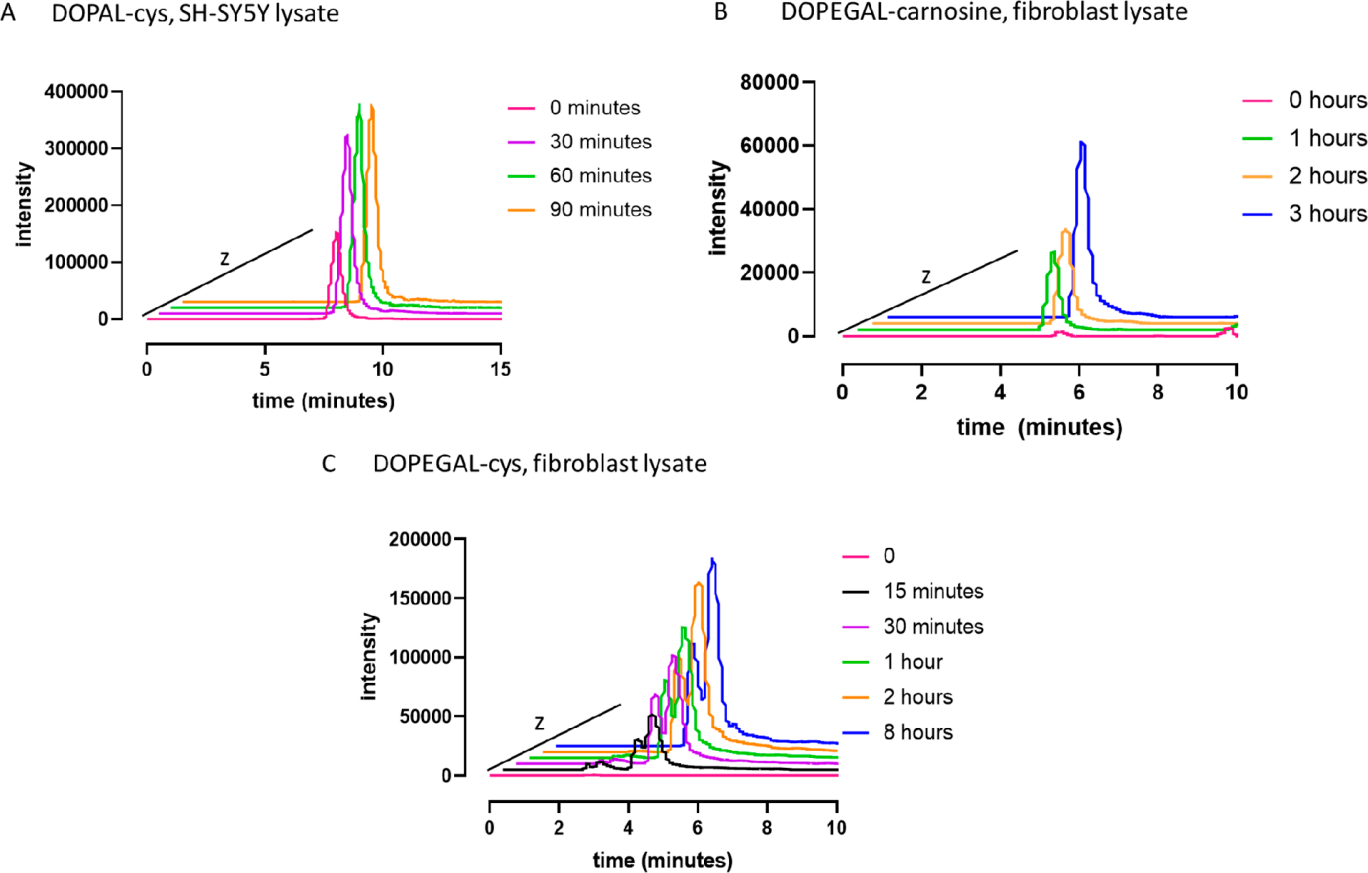
DOPEGAL and DOPAL react with carnosine and/or l-cysteine in a time-dependent manner in a biologically relevant matrix. Please note that data points were offset into the *z* axis for clarity; retention times remain consistent for each respective conjugate. (A) Extracted ion chromatogram (EIC) of DOPAL–cys (256.0638 ± 10 ppm) adduct in SH-SY5Y cell lysate. DOPAL and l-cysteine were incubated in SH-SY5Y lysate, pH = 7.4. A time-dependent formation of the DOPAL–cys conjugate can be observed in the biologically relevant matrix. (B) EIC of DOPEGAL–carnosine (377.1445 ± 10 ppm) in fibroblast lysate. DOPEGAL and carnosine were incubated in fibroblast lysate, pH = 7.4. A time-dependent formation of the DOPEGAL–carnosine conjugate can be observed in the biologically relevant matrix. (C) EIC of DOPEGAL–cys (254.0482 ± 10 ppm) in fibroblast lysate. 254.0482 *m*/*z* was chosen because it is observed in greater abundance than 272.0587 *m*/*z*. DOPEGAL and l-cysteine were incubated in fibroblast lysate, pH = 7.4. A time-dependent formation of the DOPEGAL–cys conjugate can be observed in the biologically relevant matrix. The split peak is consistent with two peaks observed in the HPLC-PDA analysis (Figure 3C).

Figure 6 reports the extracted ion chromatograms for our compounds of interest within the biological matrix. A time-dependent formation for DOPAL–cys (Figure 6A), DOPEGAL-carnosine (Figure 6B), and DOPEGAL–cys (Figure 6C) can be observed. The dehydrated molecular ion is used to represent DOPEGAL–cys as it is the most abundant peak in the full scan analysis (Figure 6E). In this representation, two nearly resolved peaks are observable. This is consistent with our HPLC-PDA data that demonstrated the formation of at least two new peaks after reaction of DOPEGAL with l-cysteine (Figure 6C); it is also consistent with the data reported by Wanner et al.^21^ Because the extracted ion chromatogram of the DOPEGAL–cys full molecular ion (272.0587 *m*/*z*) has only one resolved peak (data not shown), it is conceivable that the two peaks arise from the thiazolidine conjugate and its dehydrated product. However, the full scan of our biochemical reaction also contained peaks consistent with the hypothetical mass of other potential DOPEGAL–cys adducts (i.e., adducts that are not the thiazolidine), but we were unable to characterize these as they were in low abundance. Also, the DOPEGAL used in our reactions was not pure, and the presented chromatogram is data from a biological matrix. Finally, it is likely that multiple peaks may arise from the presence of diastereomers since DOPEGAL–cys contains three chiral centers. It is possible the multiple peaks arise from any number of these variables; more work is needed to form a precise hypothesis.

### MAO Activity Is Necessary for Conjugate Production

**Figure 7:**
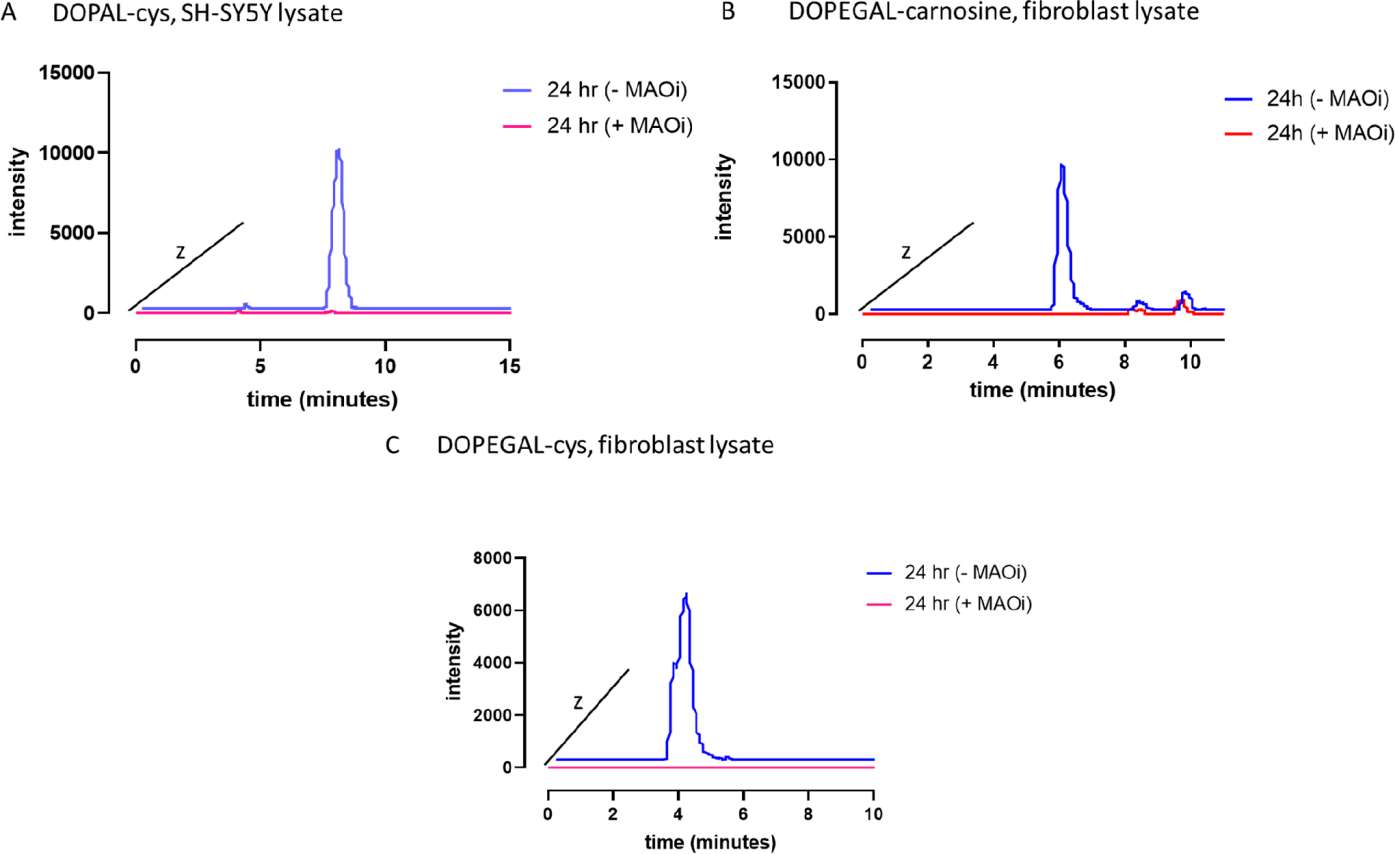
MAO inhibitors (MAOi) prevent formation of DOPAL–cys, DOPEGAL–carnosine, and DOPEGAL–cys conjugates in enzymatically active cell lysate. Please note that data points were offset into the *z* axis for clarity; retention times remain consistent for each respective conjugate. (A) EIC of DOPAL–cys (256.0638 ± 10 ppm) adduct in SH-SY5Y cell lysate. Dopamine and l-cysteine were incubated in enzymatically active SH-SY5Y lysate either in the presence (+) or absence (−) of clorgyline and selegiline, MAO-A and MAO-B inhibitors, respectively. The formation of DOPAL–cys can be observed at 24 h in the absence of MAOi. Addition of MAOi diminishes this peak. (B) EIC of DOPEGAL–carnosine (377.1445 ± 10 ppm) in fibroblast lysate. Norepinephrine and carnosine were incubated in enzymatically active fibroblast lysate either in the presence (+) or absence (−) of MAOi. The formation of DOPEGAL–carnosine can be observed at 24 h in the absence of MAOi. Addition of MAOi diminishes this peak. (C) EIC of DOPEGAL–cys (254.0482 ± 10 ppm) in fibroblast lysate; 254.0482 *m*/*z* was chosen because it is observed in greater abundance than 272.0587 *m*/*z*. Norepinephrine and l-cysteine were incubated in enzymatically active fibroblast lysate either in the presence (+) or absence (−) of MAOi. The formation of DOPEGAL–cys can be observed at 24 h in the absence of MAOi. Addition of MAOi diminishes this peak.

To determine whether these catecholaldehyde conjugates form as a result of catecholamine metabolism in a physiologically relevant system, we repeated the experiment using fresh, enzymatically active cell lysate. In this experiment, we relied on endogenous MAO in the cells to produce DOPEGAL or DOPAL via metabolism of NE and DA, respectively, and included carnosine and l-cysteine in the mixture. Conjugates were formed in a time dependent manner (data not shown) as with the inactive lysate but with extended time points to allow for metabolism. Controls revealed that neither selegiline nor clorgyline reacted with either aldehyde (data not shown). Extracted ion chromatograms are reported in Figure 7 for the 24 h time point. The experiments were done in the absence or presence of MAOi (i.e., clorgyline and selegiline to inhibit MAO-A and MAO-B, respectively). Figure 7 reports the extracted ion chromatogram of the adduct in these two conditions. Inclusion of MAOi completely abrogated the observed peaks, demonstrating that metabolism of NE or DA by MAO is necessary for conjugate production. These data further demonstrate that endogenous ALDH activity was not sufficient to metabolize the aldehydes prior to their conjugation; it is likely aldehyde conjugation was faster than aldehyde metabolism in the given conditions. In addition, ALDH and AR activity may be saturated in conditions of excess aldehyde (e.g., conditions of Parkinson’s etiology), and the cell may turn to alternative routes of detoxification.

## Discussion

The catecholaldehydes DOPAL and DOPEGAL, metabolites of DA and NE, respectively, are emerging as potentially important contributors in the pathogenesis of neurodegenerative and cardiovascular diseases.^1,3,11^ DOPAL is reported to be relevant to Parkinson’s pathogenesis due to its cytotoxicity in dopaminergic neurons, likely via its production of oxidative stress and ability to modify proteins.^6^ In the heart, where NE is abundant, DOPEGAL is suggested to play a role in cardiac injury.^11,12,24^ Both catecholaldehydes modify proteins and therefore could potentially alter protein function.^4,10^ The electrophilic nature of DOPEGAL and DOPAL is particularly interesting for two reasons. First, elucidating the reactivity of these aldehydes gives insight into their mechanisms of toxicity in cells. Second, the conjugation of DOPEGAL or DOPAL to nucleophiles could produce biomarkers indicative of disease etiology. Disruption of DA and NE metabolism pathways (e.g., ALDH disruption via pesticide exposure, as implicated in Parkinson’s disease^25,26^) likely begins years before major cell death and injury;^27^ we propose the conjugates characterized in this study may be produced during this prodromal phase, though further studies in vitro and in vivo are needed to evaluate their production and utility.

We have previously demonstrated the reactivity of DOPAL with various nucleophiles.^6,11,18^ The current study extends our previous work by expanding upon the reactivity of DOPAL and including DOPEGAL. Our research suggests that DOPAL reactivity with certain amines (carnosine and NAL) is dependent on oxidation, but that this is not the case for l-cysteine, suggesting a different mechanism of reactivity. Furthermore, we also characterize the reactivity of DOPEGAL with carnosine and l-cysteine. Our specific focus on carnosine and l-cysteine is derived from their potential to form aldehyde conjugates,^28−30^ rather than simply attenuating catecholaldehyde reactivity through antioxidant mediation. The conjugates could function as potential biomarkers for NE and DA dyshomeostasis. We were able to identify and characterize conjugates for DOPAL with l-cysteine and DOPEGAL with l-cysteine and carnosine via HPLC-MS. Note that it is difficult to conclusively state the physiologic concentrations of NE, DA, and their respective aldehydes, which vary depending on tissue location, subcellular location, and disease state, though it is known that carnosine is present in millimolar concentrations in the heart, serum, and brain tissue.^31^ Therefore, our results should be considered proof of concept; more work is needed in vitro and in vivo to conclusively demonstrate physiological viability. Furthermore, we were able to detect aldehyde conjugates via HPLC-MS with sensitivity, which is promising for translation to in vivo work.

We first investigated reactivity of the catecholaldehydes via HPLC-PDA. These data suggested that DOPAL reacts with NAL and carnosine. Our proposed mechanism, which we have previously reported, suggests Schiff base formation between the amino group (i.e., the β-alanine amino for carnosine) and the aldehyde moiety of DOPAL, followed by an oxidative activation of the catechol and rearrangement to form an indole-type structure.^19^ Our results are consistent with this hypothesized reaction. The control DOPAL decreases slightly over time, likely due to polymerization that takes place after auto-oxidation. In the presence of NAC and NAL, more DOPAL is retained, likely due to NAC preventing auto-oxidation. Similarly, reaction of NAL with DOPAL was attenuated in the presence of antioxidant NAC; NAC blocks the oxidation of the catechol and therefore prevents the formation of the stable indole product from the reversible Schiff base. In contrast, incubation of NAL with DOPAL in the presence of the reducing agent (i.e., NaCNBH_3_) slightly increased reactivity; the reduction of the Schiff base favors formation of a stable product. The same behavior was observed for carnosine. We further characterized DOPAL’s reactivity with carnosine by incubating DOPAL with the two amino acid constituents of carnosine (i.e., *N*-acetyl-l-histidine and β-alanine). Our results confirm that the β-alanine moiety (which contains the amino group) is the primary moiety involved in the reaction with DOPAL. Carnosine reactivity with DOPAL is higher than the reactivity of DOPAL with this single reactive constituent, likely due to more favorable orientation in space.

**Figure 8:**
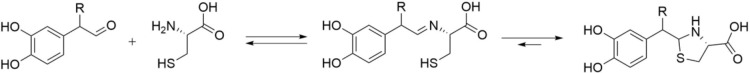
Our proposed mechanism of reactivity for DOPAL (R = H) or DOPEGAL (R = OH) with l-cysteine. The catecholaldehyde forms a Schiff base with l-cysteine, and then the thiol of l-cysteine reacts with the newly formed carbon–nitrogen double bond to produce a thiazolidine. This structure is supported by our HPLC-MS data. Furthermore, this reaction scheme does not require an oxidative activation step, consistent with our HPLC-PDA experiments.

While this HPLC-PDA data involving DOPAL supports and expands our previous work, we could not identify a DOPAL adduct with either NAL or carnosine via HPLC-MS and therefore cannot definitively confirm this proposed mechanism or product formation. We were, however, able to identify and characterize an adduct of DOPAL with l-cysteine. DOPAL–cys formation, as demonstrated via HPLC-PDA, was near instantaneous; the product peak was already observable at time ∼0. Unlike the reaction with carnosine or NAL, this product formation was not blocked by the addition of an antioxidant. Therefore, we suggest a mechanism for DOPAL–cys formation that does not require oxidative activation. We propose that the DOPAL aldehyde moiety forms a Schiff base with the amine of l-cysteine, and that the thiol group of l-cysteine then reacts with the newly formed carbon–nitrogen double bond (Figure 8). This forms a stable thiazolidine conjugate. We were able to identify this thiazolidine conjugate, with a theoretical [M + H]^+^ of 256.0638 *m*/*z*, via HPLC-MS. Fragmentation analysis of this compound was consistent with the proposed thiazolidine structure.

This is our first report also characterizing the reactivity of DOPEGAL, an aldehyde which has been far less studied as compared with DOPAL, likely due in large part to challenges with its synthesis and stability. We were able to identify and characterize DOPEGAL adducts with both carnosine and l-cysteine. DOPEGAL and carnosine seemed to create a new product via HPLC-PDA analysis, which we further confirmed by demonstrating product formation after incubation of NE, MAO-A, and carnosine. This product was not formed when an MAO-A inhibitor was present. We then characterized this adduct via HPLC-MS. The DOPEGAL–carnosine conjugate is different from the hypothesized DOPAL–carnosine reaction and structure because the hydroxyl group of DOPEGAL changes reactivity of the molecule. After Schiff base formation, the hydroxyl group introduces the possibility of a higher stability Amadori rearrangement. This hypothesized Amadori product, with a theoretical [M + H]^+^ of 377.1445 *m*/*z*, was identified via HPLC-MS. Our fragmentation analysis of the product supports this structure. However, the Amadori rearrangement does not seem to be favored for the reaction of DOPEGAL with l-cysteine. Like with DOPAL, l-cysteine seems to form a thiazolidine with DOPEGAL. Our HPLC-PDA analysis reveals two potential products, which we then characterized via HPLC-MS. We identified the theoretical [M + H]^+^ of 272.0587 *m*/*z* for the thiazolidine, as well as a dehydrated product with a difference of 18 amu. It is possible that the peak splitting observed for the thiazolidine mass is due to diastereomers as the DOPEGAL–cys conjugate contains three chiral centers. More work is necessary to confirm if the multiple peaks observed (both via HPLC-PDA and HPLC-MS) all arise from the thiazolidine, but our fragmentation analysis supports thiazolidine formation. Both structures that we suggest for the two DOPEGAL adducts have been previously hypothesized by Wanner et al.^21^ However, the products obtained in their work were formed in acidic conditions (0.1 M TFA) which are known to favor Schiff base formation. Our experiments demonstrate that these products form in buffered solution at physiological pH (7.4).

After characterization of the DOPAL–cys, DOPEGAL–cys, and DOPEGAL–carnosine conjugate, we then sought to examine if these products could form in a biological matrix where other nucleophiles compete for reaction with the catecholaldehydes. We confirmed that all three adducts form in a relevant matrix (neuronal lysate for DOPAL and fibroblast lysate for DOPEGAL). Furthermore, we demonstrated that conjugate production in a biological environment is dependent on MAO activity and that endogenous ALDH or AR activity was not sufficient to prevent aldehyde conjugate formation. In pathological conditions, oxidative stress can enhance MAO activity, while reducing the activity of ALDH and other detoxifying enzymes.^32^ These conditions create a global increase of the production and accumulation of DOPAL and DOPEGAL, which can react with proteins and induce conformational changes and loss of function.^32^ Note that these same oxidative conditions that enhance MAO activity and DOPAL and DOPEGAL production also favor the formation of quinones, which further contribute to protein modification.^6^ Oxidative stress also increases production of other reactive carbonyl species.^33,34^ The antioxidant defense in these conditions, such as glutathione action, is also reduced.^34^ Glutathione does not form a conjugate with DOPAL, but it can attenuate its reactivity.^13,19^

To conclude, in this study, we have demonstrated the unique ability of carnosine and l-cysteine to sequester catecholaldehydes through adduct formation. These findings are important for several reasons. First, they suggest that l-cysteine and carnosine may represent alternative cellular catecholaldehyde detoxification pathways (e.g., as opposed to ALDH or glutathione detoxification). Furthermore, these identifiable and characterizable conjugates could represent biomarkers of catecholaminergic and/or metabolic stress, areas of intense clinical interest given the emerging evidence in support of a pathogenic role for catecholaldehydes in neurodegenerative and cardiometabolic disease. Finally, there are undeniable pharmacological implications for the catecholaldehyde scavenging effects of l-cysteine and carnosine in the treatment of these diseases. Future studies, many of which are ongoing in our laboratories, will define the metabolic determinants of conjugate production in vivo as well as the pharmacotherapeutic effects.

## Abbreviations

ALDH: aldehyde dehydrogenase
AR: aldose reductase
DA: dopamine
DOPAL: 3,4-dihydroxyphenylacetaldehyde
DOPEGAL: 3,4,-dihydroxyphenylglycolaldehyde
EIC: extracted ion chromatogram
MAO: monoamine oxidase
MAOi: monoamine oxidase inhibitor
NAC: *N*-acetyl-l-cysteine
NAL: *N*-acetyl-l-lysine
NE: norepinephrine
PDA: photo diode array
